# Association of adverse childhood experiences (ACEs) with obesity and underweight in children

**DOI:** 10.1007/s40519-021-01314-1

**Published:** 2021-10-18

**Authors:** Tomasz Hanć, Ewa Bryl, Paula Szcześniewska, Agata Dutkiewicz, Aneta R. Borkowska, Elżbieta Paszyńska, Agnieszka Słopień, Monika Dmitrzak-Węglarz

**Affiliations:** 1 Institute of Human Biology and Evolution, Faculty of Biology, Adam Mickiewicz University, ul. Uniwersytetu Poznańskiego 6, 61-614 Poznań, Poland; 2 Department of Child and Adolescent Psychiatry, Poznan University of Medical Sciences, Ul. Szpitalna 27/33, 60-572 Poznań, Poland; 3 Faculty of Education and Psychology, Maria Curie-Sklodowska University, Ul. Prezydenta Gabriela Narutowicza 12, 20-400 Lublin, Poland; 4 Department of Integrated Dentistry, Poznan University of Medical Sciences, Ul. Bukowska 70, 60-812 Poznań, Poland; 5 Psychiatric Genetics Unit, Department of Psychiatry, Poznan University of Medical Sciences, Ul. Rokietnicka 8, 60-806 Poznań, Poland

**Keywords:** Adverse childhood experiences, Underweight, Overweight, Obesity, Parental education, Socioeconomic status, Children

## Abstract

**Purpose:**

The aim of the study was to test the hypothesis that adverse childhood experiences (ACEs) are related to both obesity and underweight from childhood, and that the association of ACEs with weight abnormalities is modulated by type of ACEs, sex and socioeconomic status (SES) indices.

**Methods:**

The relations between ACEs (0 vs ≥ 1), ACE accumulation and ACE type with weight status and *z* scores BMI were assessed in 503 children aged 6–12 years from Poznan, Poland. The effects of interaction of ACEs with sex and SES on *z* scores BMI were included in the analyses.

**Results:**

ACEs were significantly related to both obesity and underweight, in unadjusted analysis, and when sex and SES indices, such as size of place of residence, people per room in household, and parental education were controlled. The relation of ACEs with *z* scores BMI was modulated by ACE type, parental subjective assessment of economic situation of a family and parental education. ACE accumulation was not related to an increase of obesity or underweight rate, or *z* scores BMI.

**Conclusion:**

The study implicates the need for both obesity and underweight prevention in individuals with adverse experiences as early as in childhood.

**Level of evidence:**

III: evidence obtained from well-designed cohort study.

## Background

Over the last decade research has revealed an association of obesity with psychosocial determinants, e.g.: adverse childhood experiences (ACEs). Examples of ACEs are childhood maltreatment, being a victim or witness of violence, parental divorce, death or disease of a family member, family problems [1]. Numerous studies have found that the risk of obesity in adults is significantly related to adversity in earlier phases of an individual’s development [2–5]. A number of potential biosocial mechanisms for the association of ACEs with obesity have been proposed. The results of previous research suggest an important role of stress-induced changes of regulation in the hypothalamic–pituitary–adrenal axis, autonomic nervous system and prefrontal cortex, expressed in behavioural effects [6]. Nevertheless, the mechanisms underlying the ACEs-obesity link require further research.

Although a relationship between ACEs and body size has been found in many studies, there are two aspects of this link that require further investigation. First, as shown in the meta-analysis of Danese and Tan [7], the association between childhood maltreatment and obesity was best documented in adults, while studies of children and adolescents gave contrasting findings. The review of studies on the relationship between accumulation of ACEs and overweight indices in children gave significant results, nevertheless, no association was found for the most recent ACEs in children’s lives and their excessive weight [1]. Recently published systematic review suggests that some incubation period may occur before the ACEs related tendency for obesity emerges and the outcomes may vary dependent on the type of ACEs and other factors, such as sex [8, 9]. Therefore, the results on the link between ACEs and childhood obesity are inconsistent. Second, studies including possible ACE-underweight association are sparse and have produced contrasting results. The most recent research on a large sample of adolescents from the USA revealed a number of ACEs related to increasing risk of obesity but not underweight [6]. Other studies suggest that results may be diversified by sex, socioeconomic status and type of ACE. Girls may be more sensitive to the obesity-related effects of ACEs than boys [8]. One study on adults found ACEs related to obesity in women and to underweight in men [10]. Soares et al. [11] examined two birth cohorts within contrasting socioeconomic contexts: the United Kingdom and Brazil. The research showed that ACEs were related to adiposity indices in the UK adolescents, but in the Brazilian cohort ACEs were rather associated with lower waist circumference and BMI. In Danish military veterans sexual abuse in childhood was related to obesity, and emotional abuse to underweight [12]. In another study, the association of sexual abuse with obesity was confirmed in a large group of Finnish adolescents, whereas, parental unemployment was related to both obesity and underweight [13]. Finally, research on Polish children and adolescents found parental addiction to alcohol associated with underweight but not obesity [14]. These ambiguous results justify further research on the link between ACEs and both obesity and underweight, with the inclusion of the role of possible modulating factors.

The aim of the study was to assess the relationship between ACEs, BMI, and weight status in Polish children. We hypothesised that ACEs are related to both obesity and underweight from childhood, and the association of ACEs with weight abnormalities is modulated by ACE type, sex and socioeconomic status indices.

## Materials and methods

### Ethical statements and basic information about procedure

The study was funded by the National Science Centre, Poland, grant number: 2016/21/B/NZ5/00492. The work described has been carried out in accordance with The Code of Ethics of the World Medical Association (Declaration of Helsinki) for experiments involving humans and approved by the Institutional Bioethics Board of Poznan University of Medical Sciences (approval no. 542/14). The subjects and their legal guardians were fully informed about the research procedures and legal guardians gave a written consent to participate in the study. The only inclusion criteria were the age between 6 and 12 years and being a student of elementary school located in Poznań. There were no exclusion criteria. Poznań is one of the largest cities in Poland and the administrative capital of the province called Greater Poland Voivodeship located in west-central region of the country. The children were recruited in 11 randomly selected elementary schools. During meetings with parents at school, we presented the purpose and plan of the study. Parents interested in the research received detailed information about the study, an informed consent card and questionnaires. Data on adverse childhood experiences, height and weight measurements as well as socioeconomic (SES) factors were collected for each child.

### Adverse childhood experiences (ACEs) assessment

ACEs were assessed by questions designed based on the Traumatic Events Screening Inventory-Parent Report Revised (TESI-PRR) [15]. The original TESI-PRR interview consists of 24 items covering a variety of potential traumatic events including current and previous injuries, hospitalizations, domestic violence, community violence, disasters, accidents, physical abuse, and sexual abuse. We decided to use simplified list of questions and focus rather on 7 broader ACE types addressed in the TESI-PRR. The reasoning for this procedure was to limit the risk related with the study for potentially vulnerable families and make the questions more readable in a paper–pencil form for a broad class of parents. Parents of children from the sample were asked to mark all types of events experienced by their child: (1) The life or health of the child was threatened, (2) The child experienced an event in which the life or health of another person was endangered or someone died, (3) The child was physically (e.g. hitting, pushing, choking, shaking, biting, burning, forced into any type of sexual activity) or psychologically (e.g. calling him/her in an unpleasant way, mocking, gossiping, shouting at him/her very hard, threatening him, the child felt rejected by someone close to him) assaulted, (4) The child was a witness of physical or psychological assault, (5) The child experienced the death of someone close to him/her, (6) The child experienced serious family problems (e.g. quarrels, conflicts, fighting, parting, alcohol problems or other addiction types, emotional or mental problems of family members), (7) The child was separated from his/her parents for many days. These experiences are hereinafter referred to in the article as ‘life/health threat’, ‘life/health threat witness’, ‘violence victim’, ‘violence witness’, ‘death of someone close’, ‘stressful family problems’ and ‘long separation from parents’. As the list might not contain all possible adverse experiences of the studied children, the additional class of ACEs was included in the questionnaire: (8) other, not mentioned above, hereinafter referred to in the article as ‘other unspecified’ ACEs. The accumulation of adverse experiences was assessed as a sum of ACE types (ACE score) in a child’s life. ACE scores were coded as 0, 1, 2, 3 and 4+ groupings consistent with previous research [4, 16].

### Anthropometric assessment and body weight classification

Measurement of body height was carried out with the anthropometer Seca 213, with a measurement accuracy of ± 1 mm. Body weight was measured with a medical scale (TANITA MC-780) and a measurement accuracy of ± 100 g. Measurements were performed by trained medical personnel. Children were measured in light clothes, between 8 am and 2 pm. Body Mass Index (BMI) was calculated on the basis of height and weight measurements. Effects of sex and age on BMI were controlled by transformation BMI into *z* scores on the basis of World Health Organisation (WHO) growth charts [17] with the use of WHO AnthroPlus software. Underweight, overweight and obesity were diagnosed by an adequate classification of BMI under the guidance of the International Obesity Task-Force [18, 19].

### Characteristics of SES

Questions concerning SES characteristics were included in the questionnaire for parents. The size of place of residence was assessed using the number of residents and divided into three categories for the purpose of statistical analysis: village, small and medium size cities (10–100 thous. residents), large city (> 100 thous. residents). The level of parents’ education was assessed based on their level of formal education and grouped as follows: primary and vocational, secondary, higher (bachelor or master of science degree). Because such factors as significant burden on income taxes and value added tax and social security contributions the rate of people working in the grey zone in Poland was high in the time the research was conducted [20, 21]. The direct question on income level could therefore result in increased rate of dropouts from the study and heightened the risk of collecting false data. Based on this assumption we used other measures of material status. The economic situation of a family was assessed subjectively by parents and grouped into three categories: bad, average, good, and objectively, by the people per room indicator, grouped as follows: ≥ 2, 1–2, ≤ 1. The people per room indicator is widely used measure of household density that was found to be related significantly to income and socioeconomic status in previous research [22].

### Statistical methods

The analyses were performed for the individuals with complete information on ACEs and weight status. Although in several cases parents did not respond to questions concerning socioeconomic characteristics of the family, they were not excluded from the sample.

The size and significance of the effects of ACEs (at least 1 type of ACEs vs 0, or ACE scores vs 0, or ACE types vs 0) and other independent variables: sex (with boys as the reference), size of place of residence (city > 100 thous. residents as the reference), level of parents’ education (higher education as the reference), parental subjective assessment of economic situation of a family (“good” as the reference) and people per room (≤ 1 as the reference) on underweight (1-underweight vs. 0-normal weight), overweight (1-overweight vs. 0-normal weight) and obesity rates (1-obesity vs. 0-normal weight) were assessed in both unadjusted and adjusted logistic regression analysis. In adjusted analyses, the effects of ACEs-related variables were controlled for all other independent variables. Adjusted odds ratio for sex and socioeconomic indices were pooled out from the analysis including “at least 1 type of ACE vs. 0” variable and all other independent variables.

We used two-way ANOVA to assess the effects of interaction between sex, SES indicators and ACEs on *z* scores BMI. The analysis was applied for ACEs as a nominal variable (at least 1 type of ACE vs 0) and for the types of ACE for which unadjusted or adjusted logistic regression analysis showed association with both underweight and overweight or obesity. ANOVA and Pearson’s correlation analysis were applied to assess the relationship of ACE score with *z* scores BMI. The size of effects was assessed with Cohen’s *d*.

All tests were performed with Statistica (Version 12) software and considered to be statistically significant at *p* < 0.05.

## Results

### Characteristics of the sample

The sample comprised 503 children at age of 6–12 years (mean = 8.98, SD = 1.30) with a comparable proportion of boys and girls (52.49 and 47.51%, respectively) and with complete information on ACEs and weight status. Small rate of missing data occurred in case of other variables, such as size of place of residence (*n* = 16, 3.18%), parents’ level of education (mothers’ education: *n* = 7, 1.39% or fathers’ education: *n* = 23, 4.57%), parental subjective assessment of economic situation of a family (*n* = 17, 3.38%) or people per room (*n* = 6, 1.19%).

The majority of the sample consisted of residents of large city (79.47%), individuals whose parents declared ‘good’ family economic situation, higher education of mother (65.32%) and father (48.17%) living in a household of ≤ 1 people per room (41.45%). Almost half of the sample had experienced at least 1 type of ACE (47.51%), the death of close person being the most frequent (18.33%). Ten percent of the sample was classified as underweight (10.14%), 15.91% as overweight and 4.57% as obese. The basic characteristics of the sample are presented in Table1.

**Table 1:** Basic statistics by weight status

	Full sample	*z*BMI, mean (SD)	Underweight	Overweight	Obesity
Age, mean (SD)	8.98 (1.30)		8.95 (1.30)	9.11 (1.27)	8.98 (1.31)
Sex, *n* (%)					
Boys	264 (52.49)	0.40 (1.28)	22 (8.33)	45 (17.05)	12 (4.55)
Girls	239 (47.51)	0.25 (1.13)	29 (12.13)	35 (14.64)	13 (5.44)
Place of residence *n* (%)					
Villages	40 (8.21)	0.03 (1.18)	8 (20.00)	4 (10.00)	1 (2.50)
Small and medium size city (10–100 thous. residents)	60 (12.32)	0.43 (1.40)	4 (6.67)	9 (15.00)	5 (8.33)
Large city (> 100 thous. residents)	387 (79.47)	0.33 (1.18)	38 (9.82)	61 (15.76)	18 (4.65)
Parental subjective assessment of economic situation of a family, *n* (%)					
Bad	17 (3.50)	0.70 (1.50)	1 (5.88)	3 (17.65)	2 (11.76)
Average	312 (64.20)	0.34 (1.23)	32 (10.26)	53 (16.99)	16 (5.13)
Good	157 (32.30)	0.23 (1.17)	18 (11.46)	19 (12.10)	7 (4.46)
People per room, *n* (%)					
≥ 2	100 (20.12)	0.43 (1.35)	11 (11.00)	17 (17.00)	8 (8.00)
> 1 and < 2	191 (38.43)	0.30 (1.25)	20 (10.47)	34 (17.80)	11 (5.67)
≤ 1	206 (41.45)	0.28 (1.11)	20 (9.71)	26 (12.62)	6 (2.91)
Education of mother, *n* (%)					
Primary	4 (0.81)	0.85 (1.48)	0	1 (25.00)	1 (25.00)
Vocational	48 (9.68)	0.97 (1.49)	2 (4.17)	11 (22.92)	9 (18.75)
Secondary	120 (24.19)	0.45 (1.41)	14 (11.67)	22 (18.22)	10 (8.33)
Higher	324 (65.32)	0.20 (1.05)	33 (10.19)	46 (14.20)	5 (1.54)
Education of father, *n* (%)					
Primary	15 (3.13)	0.70 (1.46)	1 (6.67)	2 (13.33)	2 (13.33)
Vocational	92 (19.17)	0.77 (1.29)	5 (5.43)	28 (30.43)	8 (8.70)
Secondary	137 (28.54)	0.44 (1.24)	11 (8.03)	21 (15.33)	9 (6.57)
Higher	236 (48.17)	0.11 (1.05)	29 (12.29)	27 (11.44)	4 (1.69)
ACE, *n* (%)					
0	264 (52.49)	0.30 (1.06)	17 (6.44)	42 (15.91)	5 (1.89)
At least 1 type	239 (47.51)	0.36 (1.37)	34 (14.23)	38 (15.90)	20 (8.37)
ACE score, *n* (%)					
1	120 (23.86)	0.45 (1.31)	16 (13.33)	19 (15.83)	11 (9.17)
2	64 (12.72)	0.36 (1.57)	12 (18.75)	9 (14.06)	8 (12.50)
3	25 (4.97)	0.22 (1.15)	3 (12.00)	5 (20.00)	0
4	19 (3.78)	0.31 (1.26)	2 (10.53)	4 (21.05)	0
5	6 (0.80)	0.42 (1.46)	0	1 (15.67)	1 (16.67)
6	4 (0.80)	− 0.88 (1.43)	1 (25.00)	0	0
7	1 (0.20)	− 0.48	0	0	0
8	0		–	–	–
Type of ACE					
Life/health threat	43 (8.55)	0.11 (1.21)	6 (13.95)	5 (11.62)	2 (4.65)
Life/health threat witness	38 (7.55)	0.51 (0.97)	1 (2.63)	4 (10.53)	2 (5.26)
Violence victim	45 (8.95)	0.11 (1.51)	8 (17.78)	5 (11.11)	4 (8.89)
Violence witness	35 (6.99)	0.30 (1.38)	3 (8.57)	7 (20.00)	2 (5.71)
Death of someone close	92 (18.33)	0.26 (1.30)	12 (13.04)	13 (14.13)	5 (5.43)
Stressful family problems	90 (17.93)	0.33 (1.43)	14 (15.56)	14 (15.56)	9 (10.00)
Long separation from parents	69 (13.77)	0.27 (1.43)	11 (15.94)	11 (15.94)	6 (8.70)
Other unspecified	24 (4.87)	0.34 (1.52)	4 (16.67)	7 (29.17)	1 (4.17)
*N* (% of full sample)	503		51 (10.14)	80 (15.91)	25 (4.97)

*ACEs* adverse childhood experiences, *zBMI* *z* scores for BMI, *n* number, *%* percent, *SD* standard deviation

### The effects of ACEs, sex and SES on weight status

Unadjusted logistic regression analysis revealed associations of ACEs (≥ 1 vs 0) with both underweight (OR = 2.93, 95%CI 1.56–5.52) and obesity (OR = 5.44, 95%CI 1.99–14.87). Similar results were found for ACE types: violence victim, stressful family problems and long separation from parents. Other unspecified ACEs were related to underweight and overweight, and the death of someone close only with underweight. The analyses adjusted for other variables confirmed the association of ACEs (≥ 1 vs 0), and specifically of ACE types, such as violence victim and stressful family problems, with underweight and obesity. Long separation from parents remained significantly related to underweight, and other unspecified ACEs to overweight. The association of the death of someone close with underweight lost statistical significance in adjusted analysis. When effects of ACE accumulation were tested, both underweight and obesity were significantly related only with one type of adverse experiences or accumulation of two ACE types in adjusted but not in unadjusted analysis. Further accumulation of 3 or ≥ 4 ACE types were not associated with weight abnormalities. The results did not indicate that the accumulation/dose of ACEs is related to higher risk of underweight or obesity comparing to a single adverse experience (Table 2).

**Table 2:** The logistic regression analysis’ results for weight status as dependent variables and ACEs, sex and socioeconomic status as predictors

	Unadjusted analyses*			Analyses adjusted for all other variables*		
	Underweight	Overweight	Obesity	Underweight	Overweight	Obesity
	OR (95% CI)	OR (95% CI)	OR (95% CI)	OR (95% CI)	OR (95% CI)	OR (95% CI)
Sex (Reference: boys)	1.58 (0.86–2.88	0.89 (0.54–1.45)	1.24 (0.55–2.79)	1.54 (0.78–3.02)	1.01 (0.60–1.69)	1.76 (0.67–4.62)
Place of residence (Reference: city > 100 thous. residents as the reference)	1.32 (0.86–2.04)	0.85 (0.54–1.34)	1.02 (0.51–2.03)	1.26 (0.77–2.06)	0.83 (0.51–1.35)	1.09 (0.45–2.61)
Parental subjective assessment of economic situation of a family (Reference: ‘good’)	0.88 (0.50–1.54)	1.40 (0.86–2.29)	1.46 (0.67–3.19	0.66 (0.34–1.30)	1.33 (0.77–2.32)	0.57 (0.22–1.50)
People per room (Reference: ≤ 1 person/ room)	1.14 (0.77–1.69)	1.29 (0.93–1.78)	**1.78 (1.05–3.01)**	1.05 (0.64–1.70)	0.95 (0.65–1.38)	1.29 (0.67–2.48)
Education of mother (Reference: ‘higher education’)	0.94 (0.57–1.54)	**1.51 (1.07–2.12)**	**4.02 (2.36–6.85)**	1.12 (0.53–2.38)	1.01 (0.63–1.60)	**4.02 (1.87–8.66)**
Education of father (Reference: ‘higher education’)	0.74 (0.47–1.15)	**1.78 (1.31–2.41)**	**2.54 (1.48–4.36)**	0.75 (0.42–1.33)	**1.60 (1.09–2.36)**	1.10 (0.53–2.28)
At least 1 type of ACE (Reference: 0)	**2.93 (1.56–5.52)**	1.23 (0.75–2.01)	**5.44 (1.99–14.87)**	**3.48 (1.70–7.10)**	1.14 (0.66–1.99)	**5.46 (1.80–16.56)**
ACE score (Reference: 0)						
1	1.62 (0.82–3.24)	1.03 (0.56–1.88)	3.12 (0.84–11.57)	**3.27 (1.44–7.41)**	1.24 (0.63–2.43)	**6.61 (2.05–21.37)**
2	1.14 (0.77–1.70)	1.00 (0.71–1.39)	1.81 (0.83–3.94)	**2.24 (1.39–3.63)**	0.97 (0.60–1.57)	**2.60 (1.21–5.57)**
3	1.08 (0.73–1.59)	1.02 (0.71–1.47)	-	1.38 (0.84–2.26)	1.10 (0.74–1.62)	-
4 +	0.94 (0.54–1.63)	1.05 (0.62–1.79)	1.33 (0.62–2.83)	1.00 (0.61–1.63)	1.05 (0.79–1.39)	1.06 (0.58–1.92)
Type of ACE (Reference: 0—no ACE reported)						
Life/health threat	2.67 (0.96–7.45)	0.79 (0.29–2.17)	2.67 (0.49–14.47)	2.62 (0.81–8.55)	0.91 (0.31–2.62)	1.51 (0.15–15.60)
Life/health threat witness	0.40 (0.05–3.19)	0.61 (0.20–1.84)	2.58 (0.48–13.99)	1.40 (0.15–13.55)	0.52 (0.16–1.70)	1.00 (0.09–10.52)
Violence victim	**3.57 (1.39–9.15)**	0.85 (0.31–2.34)	**5.71 (1.44–22.68)**	**4.92 (1.63–14.82)**	0.98 (0.34–2.87)	**6.31 (1.20–33.21)**
Violence witness	1.63 (0.44–6.06)	1.45 (0.58–3.61)	3.48 (0.63–19.10)	0.71 (0.07–6.76)	1.63 (0.59–4.50)	3.92 (0.59–26.23)
Death of someone close	**2.46 (1.10–5.50)**	1.00 (0.50–1.98)	3.23 (0.90–11.56)	2.52 (0.98–6.47)	0.99 (0.46–2.16)	3.07 (0.69–13.57)
Stressful family problems	**6.79 (2.17–21.21)**	1.26 (0.64–2.48)	**6.79 (2.17–21.21)**	**5.10 (1.86–14.00)**	1.15 (0.52–2.58)	**6.00 (1.46–24.65)**
Long separation from parents	**3.35 (1.45–7.78)**	1.28 (0.60–2.70)	**5.85 (1.70–20.19)**	**3.91 (1.48–10.32)**	1.06 (0.44–2.53)	2.96 (0.63–13.83)
Other unspecified	**4.17 (1.20–14.50)**	**2.78 (1.03–7.51)**	3.33 (0.36–31.15)	4.93 (0.96–25.40)	**3.62 (1.11–11.79)**	2.08 (0.15–28.89)

*Incidence of underweight, overweight, obesity were tested against the reference—normal weight

*ACEs *adverse childhood experiences, *OR* odds ratio, *CI* confidence interval, *bold* the relation is statistically significant at the level of < 0.05

Low education of mother and father as well as > 1 people per room were associated with excess weight in unadjusted analyses. The relation remained significant for education of mother (OR = 4.02, 95%CI 1.87–8.66 for obesity) and father (OR = 1.60, 95%CI 1.09–2.36 for overweight) but not for people per room in adjusted analyses. Abnormal weight was not related to child’s sex, place of residence or parental subjective assessment of SES.

### The effects of ACEs, sex and SES interactions on *z* scores BMI

**Fig. 1:**
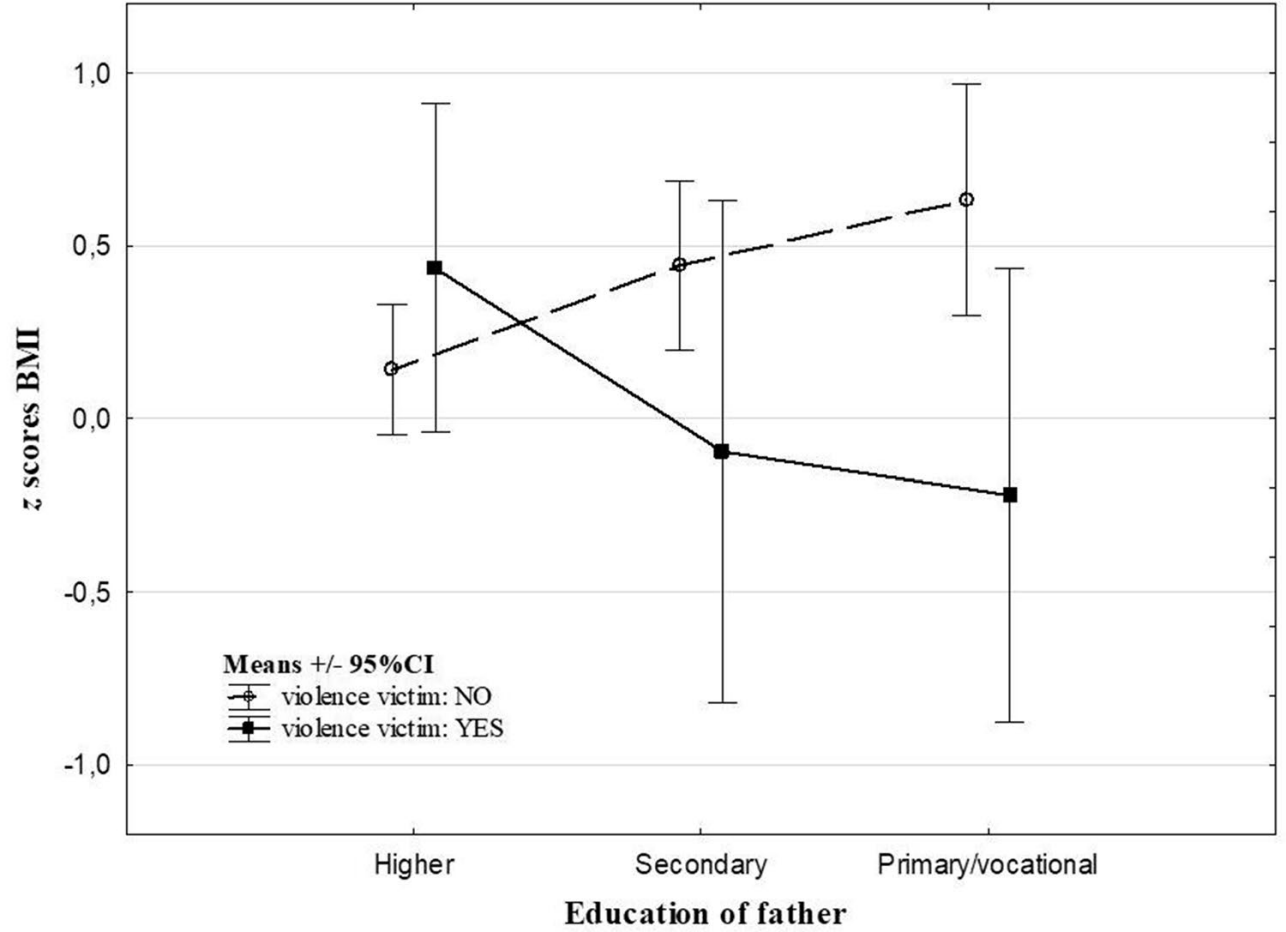
The effects of interaction between an education of father and the experience of being victim of violence on the *z* scores BMI

**Fig. 2:**
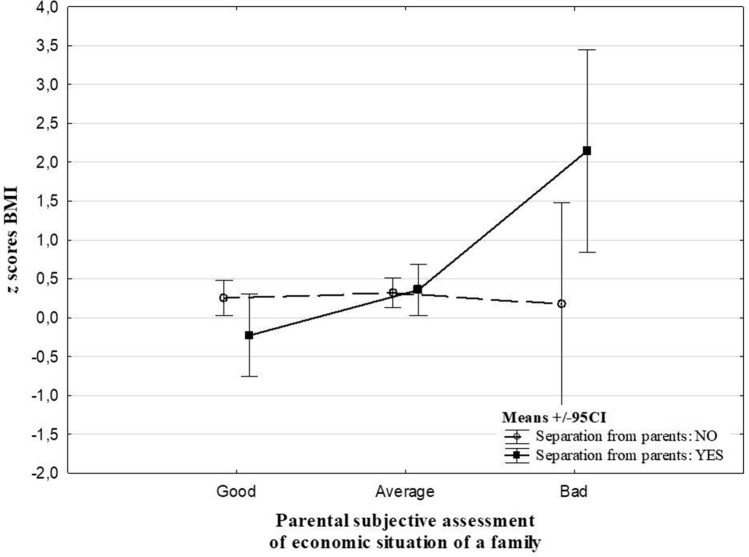
The effects of interaction between parental subjective assessment of socioeconomic status of the family and the long separation from parents on the *z* scores BMI

**Fig. 3:**
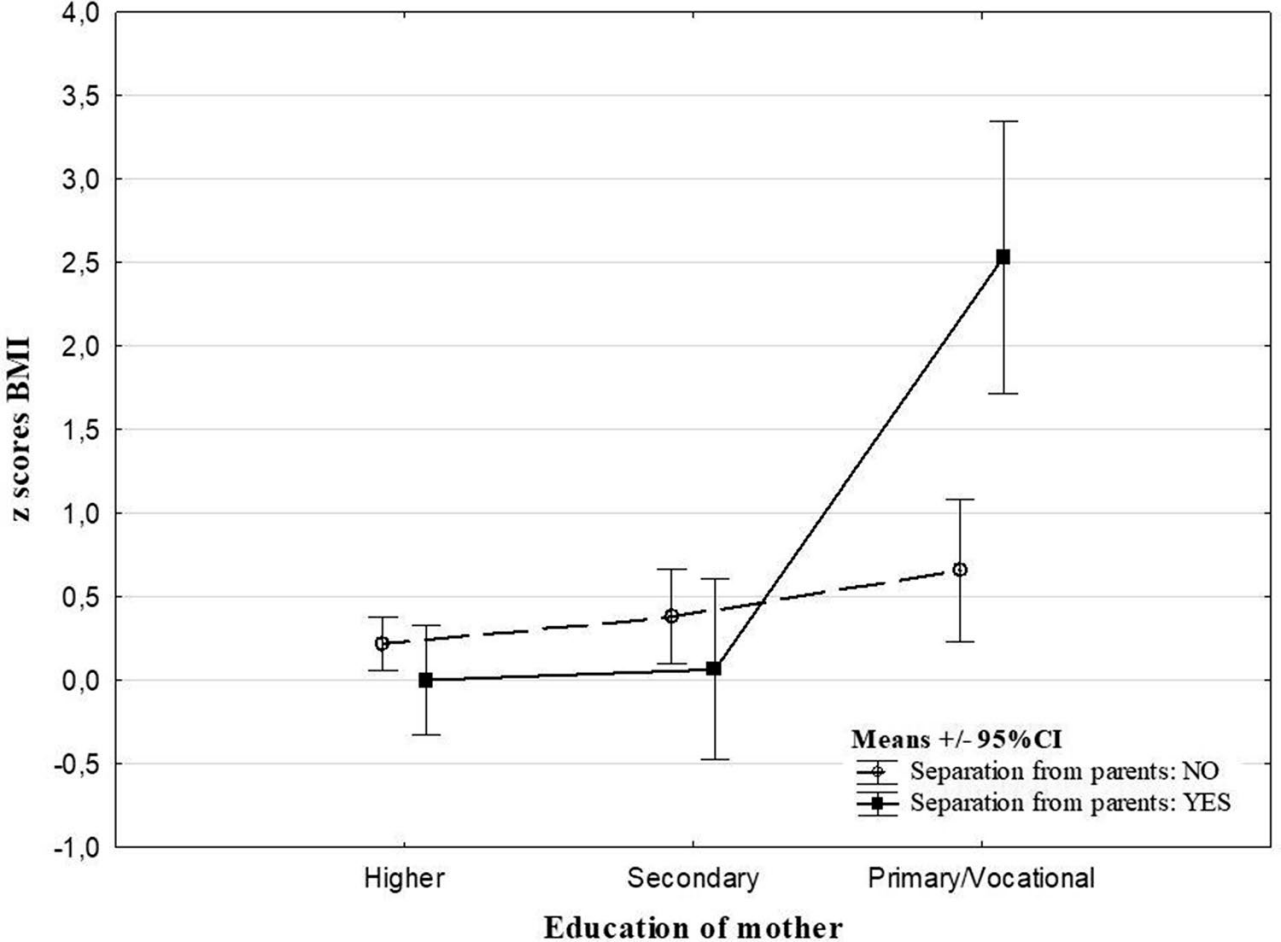
The effects of interaction between an education of mother and the long separation from parents on the *z* scores BMI

The analysis revealed effects of interaction between parental subjective assessment of the economic situation of the family, education of parents and ACE types on *z* scores BMI (Table 3). Experience of violence was related to decreased *z* scores BMI (− 0.22 vs 0.63, Cohen’s *d* = 0.65) in children whose fathers’ education was ‘primary/ vocational’ (Fig. 1). Separation from parents was related to an increase of *z* scores BMI in children whose parents assessed the economic situation of the family as ‘bad’ (2.15 vs 0.18, Cohen’s *d* = 1.39) and whose mothers’ education was primary or vocational (2.53 vs. 0.66, Cohen’s *d* = 1.95) in comparison to children with no ACEs (Figs. 2 and 3). There were no significant interaction effects of ACEs and sex, places of residence and people per room on *z* scores BMI.

**Table 3:** The main effects of ACEs and interactions between ACEs and socioeconomic factors from the two-way ANOVA

	Main effects of ACEs		Interaction effects											
			Sex		Place of residence		Parental subjective assessment of economic situation of a family		People per room		Education of mother		Education of father	
	*F*	*p*	*F*	*P*	*F*	*p*	*F*	*p*	*F*	*p*	*F*	*p*	*F*	*p*
At least 1 type of ACE	0.33	0.57	0.12	0.73	0.26	0.77	0.42	0.66	0.03	0.97	1.71	0.18	0.55	0.58
Violence victim^#^	1.08	0.30	0.01	0.94	2.46	0.09	0.60	0.55	0.24	0.79	0.68	0.51	**3.71**	**0.03**
Stressful family problems^#^	0.04	0.85	0.53	0.47	1.01	0.36	0.32	0.72	0.54	0.58	1.11	0.33	0.87	0.42
Long separation from parents^#^	0.04	0.85	0.002	0.97	1.85	0.16	**3.52**	**0.03**	0.54	0.58	**9.24**	** < 0.001**	1.70	0.19
Other unspecified^#^	0.03	0.86	0.04	0.85	2.68	0.07	1.91	0.15	0.89	0.41	2.69	0.07	1.58	0.21

*ACEs* adverse childhood

^#^0 (no ACE reported) vs 1 (specific ACE type occurrence in the life history), *bold* the relation is statistically significant at the level of < 0.05

**Fig. 4:**
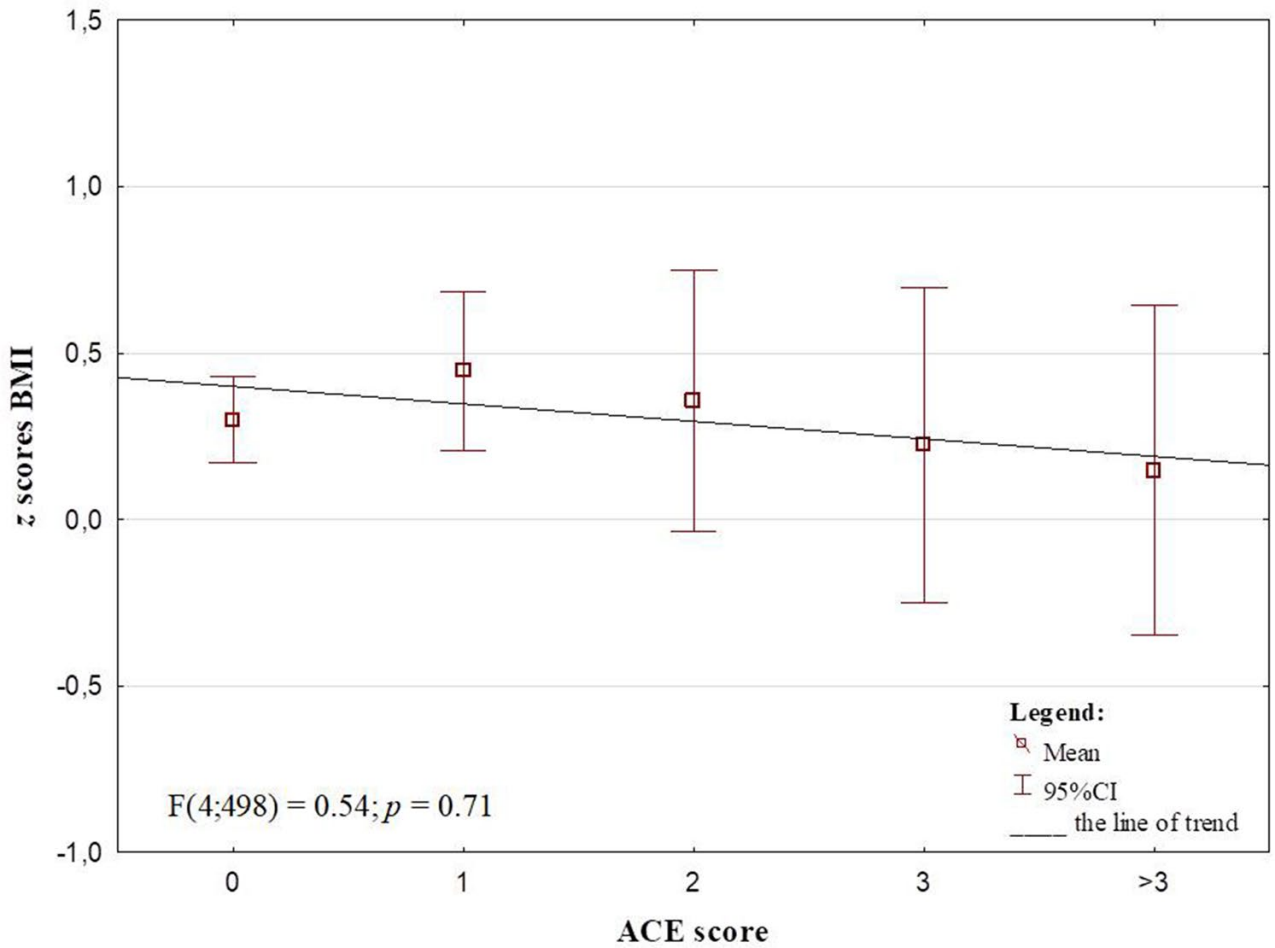
The *z* scores BMI by ACE score. The trend line and ANOVA results

A negative trend was observed in the relationship between the number of ACEs and *z* scores BMI, nevertheless the correlation was not significant (*r* = − 0.02, *p* = 0.71) and the ANOVA showed that the differences in *z* scores between the categories of ACE accumulation were not significant (Fig. 4).

## Discussion

The research revealed a high prevalence of adverse childhood experiences among the examined children and youths. Almost half of the sample had experienced at least one type of ACE. This result is consistent with other cross-sectional studies that showed a high life time frequency of traumatic stimuli in youth [6]. Taking into consideration the wide scale of the problem, there is a need for a reliable assessment of the consequences of ACEs for physical and mental health, especially in childhood and adolescence, which are periods of increased sensitivity to stress [23].

The results of the study justify the statement that the one of the earliest comorbid conditions of ACEs is an abnormal weight. It has been suggested previously that the link between ACEs and obesity may not be expressed in children because of their dependency on caregivers, also in the aspect of food consumption [24]. Contrary to this, our study confirmed a significant association between ACEs and obesity in Polish children and adolescents. This finding of the ACE-obesity link examination is consistent with relevant studies on adults [2–5] and several studies on children and adolescents [6, 13, 25, 26]. However, unlike most of the previous research [1], our research does not support a stepwise positive association between accumulation of ACEs and obesity rates. The relation was became weaker from 1 to 2 ACEs, and for > 2 ACEs it lost statistical significance. However, the summary score of the number of ACEs does not take into consideration that different ACEs may cause different psychological and physiological responses [27] and, as we have shown in the study, have varied effects on weight. The outcome of accumulation of different types of ACEs may be a compromise between an action of both mechanisms, leading to increase and decrease of the weight of a child. What is more, the study revealed that different types of ACE interact specifically with other variables. Therefore, the type of ACEs and its duration may be more predictive for weight change direction than the summary ACE score. In addition, recent studies indicate that the influence of ACEs on the development of childhood obesity may take up to 2–5 years [8]. In our study, we do not have access to information when specifically children experienced trauma and whether these were one-time events or a permanent crisis. In future studies, it is worth expanding the questionnaire to include this perspective.

The analysis of the ACE score-underweight link gave the same outcomes as in the case of obesity. Although the *z* scores BMI decreased with ACE accumulation, the trend was not statistically significant. Whereas, the study of Soares et al. [11] produced similar outcomes when the BMI and waist circumference were analysed in Brazilian adolescents, the ACE score was positively associated with obesity indices in the UK sample. The ACEs-underweight link has been revealed in several other studies [10, 12, 14]. The outcomes suggest the association of ACEs with abnormal weight is modulated by sex, socioeconomic status and ACE type. Thus, the effects of these factors on the ACEs—*z* scores BMI relation were tested in our research. Significant results were found for the interaction between long separation from parents and parental subjective assessment of SES and maternal education, with the highest *z* scores BMI in children of parents who declared the worst economic situation of family or whose mothers had finished education with a primary or vocational degree. This is in line with previous research. Despite of different method of economic situation assessment, similarities to our results were found in the work of Gardener et al. [26], which revealed an effect of interaction between income and adverse experiences on *z* scores BMI in youth. Children from low-income households are likely to experience a disproportionate burden of psychosocial stressors, such as housing or food insecurity. These adverse experiences related to economic situation were previously found to be associated with obesity [28].

A gradual increase of *z* scores BMI were found between higher and primary/vocational paternal education in children who were not violence victims. This corresponds with previous data showing the higher education of parents as a factor related to health awareness, and thus protection against obesity [29], and also underweight [30]. However, a combination of low paternal education and experience of violence was related to the lowest *z* scores BMI. This may suggest some specific characteristics of violence as one of the ACE types and/ or partially different role of paternal and maternal education. Previous study on relationship between violence and weight status has revealed contrasting findings. Whitaker et al. [31] found psychological aggression and physical punishment unrelated to obesity in preschool children, whereas, neglect was associated with obesity independently of parental education in the same sample. The results of Sokol et al. [32] suggested maltreatment in adolescence, but not in childhood, was related to excess BMI. In contrast to these studies, our results showed a significant association of experiencing violence with both underweight and obesity. However, the *z* scores BMI analysis indicated that paternal education may have a decisive influence on the type of weight abnormalities in the sample of children who experienced violence. Although both maternal and paternal education effects modulate the ACEs-weight relation, they may be related with different agents. Mothers often have a decisive effect on dietary and generally health-related habits of a family [33]. Thus, low maternal education, concomitant with a lower level of health awareness, may result in less healthy eating habits of a family and in consequence, obesity [34]. Because of the existing gender wage gap in Poland [35], fathers usually make a greater contribution to the household budget. Therefore, low parental education may affect the socio-economic status of a family to a greater extent than education of the mother. Low education of the father and low SES of a family was found previously to be related to an increased risk of different kinds of maltreatment, including violence [36, 37]. What is more, low SES and violence seem to be aggregated with food insecurity [38], which is an important risk factor not only of obesity, but also underweight [39]. This is in line with our previous study on the effects of parental alcohol addiction, which is important risk factor of child abuse and neglect [40]. The research revealed that children from fragile families characterised by parental alcohol addiction and low economic status were at high risk of underweight, and not of obesity [14]. These results suggest that under high stress conditions, and in families with very low socio-economic status, the strategy of reducing discomfort through food may be unavailable. Therefore, underweight may be the one of expected outcome in such situations.

The association of ACEs and body weight abnormalities could be explained by the development of a thrifty phenotype under the influence of adverse life events [41].The thrifty phenotype leads to changes in the child's metabolism [42], which are related with reduced energy expenditure [43], but also with greater susceptibility to the consumption of large amounts of food [44]. Overeating can be fostered by hormonal changes under the influence of stressors [45], among others. Cortisol, which promotes the deposition of fatty tissue in the abdominal area [46], which increases the risk of cardiovascular disease [47]. In addition, the increased concentration of cortisol inhibits lipolysis [48], which further reduces fat burning and promotes overweight. As shown previously, epigenetic changes have been found in several genes participating in regulation of glucose and fat metabolism [49]. The dysregulation of hypothalamic–pituitary–adrenal (HPA) and altered cortisol stress response in people who experienced ACEs may be a mediator between ACEs and failing health [50]. Thus, underweight and obesity may be the effects of different, genetically determined metabolic predispositions triggered by ACEs. Neurobiological regulation of emotions may also play an important role. Obesity was linked previously to low self-regulation, low inhibitory control skills and dysregulation in the reward pathways [28]. Early traumatic experiences can deregulate a child's developing neurobiological system, making them less resilient to stressful events [51]. ACEs can have an impairing effect on prefrontal cortex, associated with emotion self-regulation [52]. As a result, the child may engage in maladaptive coping strategies such as emotional eating [53, 54]. People who have experienced strong stressors are more prone to eating disorders [55, 56] leading to both food restriction and overeating [57]. ACEs may also be related to the increased occurrence of addictions [58], which are also associated with changes in body weight [59]. Future research on the ACEs-body mass relation should consider a closer examination of these mechanisms.

## Conclusions

The study revealed an association of adverse childhood experiences with obesity and underweight in children. The effect of ACEs on weight abnormalities was significant and distinct from sex and family characteristics. Nevertheless, the results indicate the interactions between the types of ACE, economic situation of a family and education of parents as possible modulators of ACEs-BMI association. The study implicates the need for both obesity and underweight prevention in individuals with adverse experiences as early as in childhood.

## Strength and limits

Unlike most other studies, this research tested the relation of ACEs to both obesity and underweight in children. The associations were checked for several factors in statistical analysis. What is more, the role of ACE type, sex and socioeconomic status indices as modulators of the link between ACEs and BMI was included in the analysis.

Despite the strengths of the research, there are also limitations that should be taken into consideration. The study is of a cross-sectional nature with a parental retrospective assessment of adverse childhood experiences and we did not collect any data on the height and weight of the children prior to the study. Thus, we can only conclude on the association, not causal relationship of ACEs and weight status. We cannot exclude that revealed link between ACEs and body size is partially related to greater risk of specific types of adverse experiences, e.g. peer violence, among underweight and obese children. Nevertheless, it is highly unlikely for most types of the ACE tested to be a consequence of child’s characteristics.

Parental retrospective assessment of ACEs in children may lead to underestimation, nevertheless, we decided not to question the children on ACEs directly, as potentially harmful. We also did not assess the timing and duration of exposure to ACEs. These characteristics of stress may partially explain the diversified results on ACEs-weight relation. The study was conducted on a relatively large sample, nevertheless, the low number of cases in some groups, may have resulted in insufficient statistical power and increased risk of type II error. The study was aimed to explore the interactions between ACEs, possible modulators and body size. We did not control the results of our analyses for multiple testing, therefore, type I errors cannot be excluded, either. The results obtained need to be replicated in future research.

## What is already known on this subject?

An association between adverse childhood experiences (ACEs) and obesity was found in adults, but the studies of children and adolescents gave contrasting findings. ACEs may be related not only to obesity but also to underweight. Nevertheless, studies including possible ACEs-underweight association are sparse and have produced inconsistent results.

## What this study adds?

The research presents important results on the ACEs-obesity as well as ACEs-underweight link. The associations were found to be significant in school age children. The role of such modulators as the ACE types, economic situation of a family and the level of education of parents has been revealed.

## Data Availability

Because of the sensitive nature of the data supporting the conclusions of this article, only selective access to data is offered on reasonable request to the principal investigator (MD-W).
